# Automotive Frequency Modulated Continuous Wave Radar Interference Reduction Using Per-Vehicle Chirp Sequences

**DOI:** 10.3390/s18092831

**Published:** 2018-08-27

**Authors:** Youn-Sik Son, Hyuk-Kee Sung, Seo Weon Heo

**Affiliations:** Electronics and Electrical Engineering, Hongik University, 72-1 Sangsu-Dong, Mapo-Gu, Seoul 04066, Korea; yoonsic2005@naver.com (Y.-S.S.); hksung@hongik.ac.kr (H.-K.S.)

**Keywords:** FMCW radar, automotive radar, radar mutual interference, multi-target situation

## Abstract

Recently, many automobiles adopt radar sensors to support advanced driver assistance system (ADAS) functions. As the number of vehicles with radar systems increases the probability of radar signal interference and the accompanying ghost target problems become serious. In this paper, we propose a novel algorithm where we deploy per-vehicle chirp sequence in a frequency modulated continuous wave (FMCW) radar to mitigate the vehicle-to-vehicle radar interference. We devise a chirp sequence set so that the slope of each vehicle’s chirp sequence does not overlap within the set. By assigning one of the chirp sequences to each vehicle, we mitigate the interference from the radar signals transmitted by the neighboring vehicles. We confirm the performance of the proposed method stochastically by computer simulation. The simulation results show that the detection and false alarm performance is improved significantly by the proposed method.

## 1. Introduction

Recently, interest in autonomous vehicle is increasing rapidly. The vehicles using advanced driver assistance system (ADAS) functions such as adaptive cruise control (ACC), forward collision warning (FCW), lane-keeping aid (LKA) and parking aid systems are already widely deployed on the road. To make self-driving feasible, several sensors such as the camera, lidar and radar are used. Radar sensors are especially important since, unlike other sensors, they provide exact information about the distance and speed of neighboring vehicles regardless of the weather conditions [1].

However, as the number of vehicles equipped with radar and the number of radars mounted per vehicle increases, so does the radar-to-radar interference, since the radars are concurrently operating in the same frequency band [2,3,4]. The most intuitive way to avoid this problem is to use a different frequency band, which is difficult to apply considering the wide bandwidth of the radar signal. Another method is to detect and remove the signal interference from other vehicles [5]. To determine the interference signals, the transmitter switches off the transmission signal. The problem is that it wastes the transmission opportunity during the interference measurement period and that it measures a delayed interference.

Recently, several methods have been studied to reduce interference between automotive radars. The method proposed in [6] estimates the interference signal and subtracts it from the received signal. The method of obtaining the interference signal is to obtain the minimum value of the magnitude for each phase within several discrete Fourier transform (DFT) chirps of each received signal. The problem with this method is that it requires a lot of chirp signals in the process of determining the interference signal, so it is expected to have difficulties in a fast time-varying environment. In addition, this method does not work properly if either the power of the interference signal is strong compared with the desired signal or the frequency slope difference between the desired and interferer is small. There is a way to reduce the mutual interference of automotive radar through adaptive beamforming [7]. The problem of the method proposed in [7] is that the computational complexity is significant because the adaptive weight should be obtained using the steepest-descent method. Furthermore, it needs a certain convergence time so it cannot cope well with the rapidly time-varying interferences. Due to these problems it is not easy to apply this method to autonomous vehicles. The method proposed in [8] uses morphological component analysis (MCA) to separate the interference from the received signal. The problem of this method is that the interference cannot be separated when the slope of the transmitted chirp signal and the slope of the chirp signal of interference are the same. The authors in [9] proposed a method where they apply a filter to differentiate the transmitted radar signal from the interference signals of other radars. They process the received signal iteratively until the desired signal is extracted. Their method is computationally complex and takes long time, which is not recommendable to the autonomous vehicle where the real time response is critical. There is also a method to reduce the radar-to-radar interference using the characteristics of multiple-input multiple-output (MIMO) radar [10]. It cannot be applied to the automobile radar since most of them use multi-antenna for beamforming purposes rather than MIMO purposes.

We have extensively reviewed the previous works on radar interference mitigation methods and have found that most of the methods mentioned did not consider the multi-target situation which occurs frequently in real traffic environments. Also, the fact that the power of the interference signal can vary widely is overlooked. In this paper, we propose a novel method to reduce the radar-to-radar interference in multi-target situation which works well even when the interfering signal power is high. The proposed method is based on the idea of per-vehicle transmission waveform sequence of the FMCW to reduce the radar-to-radar interference.

## 2. System Description

### 2.1. Frequency Modulated Continuous Wave Radar System

**Figure 1:**
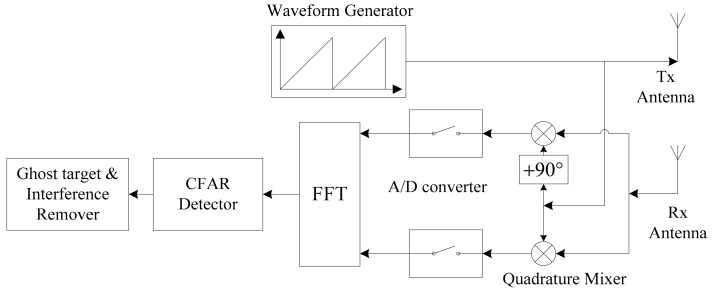
Block diagram of frequency modulated continuous wave (FMCW) radar system. FFT: fast Fourier transform; CFAR: constant false alarm rate.

In Figure 1 the frequency modulated continuous wave (FMCW) radar system block diagram is shown. The transmitter generates the FMCW waveform and the same waveform is used for the demodulation of the signal from the receiver antenna. After sampling the down-converted quadrature received signal we apply a fast Fourier transform (FFT) and process the signal in the frequency domain to remove the interference and detect the target [11,12,13].

The transmitted signal is a FMCW signal whose frequency increases linearly as given by:

(1) sT(t)=Re{ej2π(fc+a/2⋅t)t }=cos(2π(fc+a2⋅t)t),

where *f_c_* is the center frequency and *a* is the slope of the frequency domain ramp signal [4]. The received signal from several targets can be described by:

(2) $$s_{R}(t)=\sum_{m=1\;}^{N}A_{m}s\left(t-\tau_{m}\right)+w\left(t\right),$$

where *N* is number of the targets, *A_m_*, *τ_m_* are the attenuation and time delay from the *m^th^* target, respectively, and *w*(*t*) is the AWGN signal. After down-conversion we get the beat frequency signal given by:

(3) $$s_{b}(t)=\sum_{n=1\;}^{N}A_{m}e^{j2\pi (f_{b_{m}}\cdot t+\phi_{m})}+n\left(t\right),$$

where $f_{b_{m}},\;\phi_{m}$ are the beat frequency and phase between the transmitted and received signal from the target, respectively, and n(t) is the additive white Gaussian noise (AWGN) signal. The maximum beat frequency is determined by the maximum distance from the target $R_{max}$ such that $f_{b_{max}}=\frac{2R_{max}}{c}\cdot \frac{B}{T}$ where *B* is the signal bandwidth, *T* is the sweep time. Sampling frequency $F_{S}\;$ should be larger than twice the maximum beat frequency to avoid the aliasing, i.e.:

(4) $$F_{S}>2\cdot f_{b_{\max\;}}=2\cdot \frac{2R_{\max}}{c}\cdot \frac{B}{T}.$$

After the FFT operation, we apply the constant false alarm rate (CFAR) detection algorithm to determine the existence of the valid beat frequency. To decide the proper threshold value, several CFAR algorithms such as the CA-CFAR and OS-CFAR were proposed [14].

### 2.2. Multi-Target Problem

**Figure 2:**
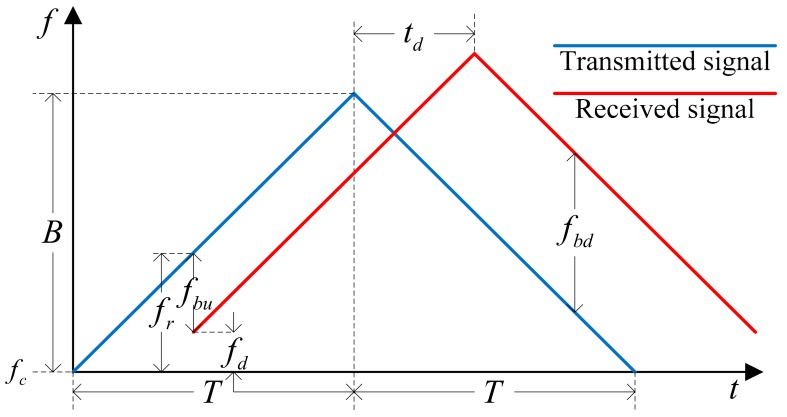
Basic waveform of FMCW radar.

The basic waveform that the FMCW radar transmits and receives is the triangle waveform and it is shown in Figure 2.

The waveform consists of an up-ramp and a down-ramp, and the beat frequencies available in each ramp are denoted by $f_{bu}$ and $f_{bd}$. The time delay between the transmitted and the received signal is denoted by $t_{d}$, and $f_{d}$ is the Doppler frequency caused by the relative velocity of the target. The bandwidth of the waveform, the period of one ramp and the carrier frequency are represented by *B*, *T* and $f_{c}$, respectively. The beat frequency of each ramp ($f_{bu}$, $f_{bd}$) can be expressed by the distance (*R*) and the relative velocity (*v*) of the target as shown by:

(5) $$\{\begin{array}{c} f_{bu\;}=f_{r}-f_{d}=t_{d}\frac{B}{T}+2\frac{v}{c}f_{c}=\frac{2R}{c}\frac{B}{T}+2\frac{v}{c}f_{c}\\ f_{bd}=f_{r}+f_{d}=t_{d}\frac{B}{T}-2\frac{v}{c}f_{c}=\frac{2R}{c}\frac{B}{T}-2\frac{v}{c}f_{c} \end{array}.$$

If where exists only a single target, we can get exact range and relative velocity of the target using Equation (5) without any problem. However, where multiple targets exist, there is a problem in obtaining the range and the relative velocity of the target through the beat frequencies.

In the multi-target situation, the process of detecting a target using FMCW radar can be divided into two steps. To ease the explanation, we assume that triangle waveform in frequency domain is transmitted. In the first step, the up and down beat frequencies ($f_{bu}$, $f_{bd}$) between the transmitted and reflected signal from the target is obtained using the CFAR algorithm. In the second step, we decide the distance (*R*) and relative velocity (*v*) of the target using the detected beat frequency pairs by the following equations:

(6) $$\{\begin{array}{c} R=-f_{c}\frac{T}{B_{1}\;}v+f_{bu}\frac{cT}{2B_{1}}\\ R=+f_{c}\frac{T}{B_{2}}v+f_{bd}\frac{cT}{2B_{2}} \end{array}.$$

**Figure 3:**
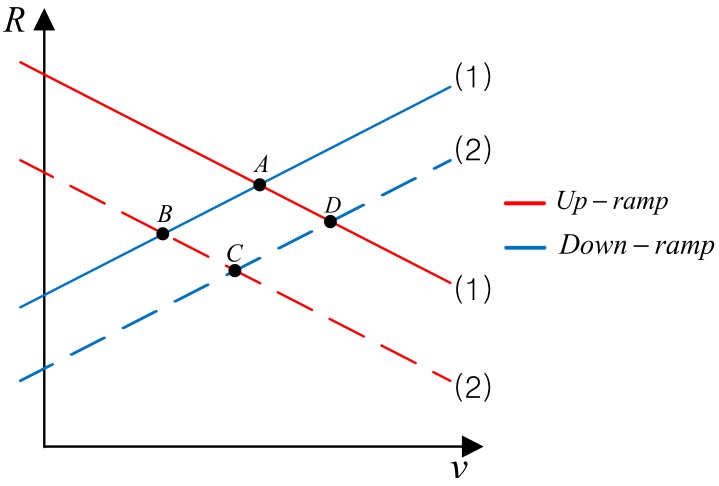
Example of multi-target problem in *R*-*v* plane.

According to the Equation (6), we get several targets from each of the combinations of the up and down beat frequency. Some combinations give a true and others give a ghost target. In Figure 3, we have shown the case where there exist two true targets. Since each target generates its own up and down beat frequency, there exists four intersection points from A to D. Among them A and C corresponds to the true target, however, the other two corresponds to the ghost target, i.e., B and D. In this case, there’s only two ghost targets. However, as the number of targets increases, otherwise as the number of $f_{bu}$ and $f_{bd}$ increases, the possible combination of the up and down beat frequency increases, the ghost-target problem becomes very difficult to solve.

**Figure 4:**
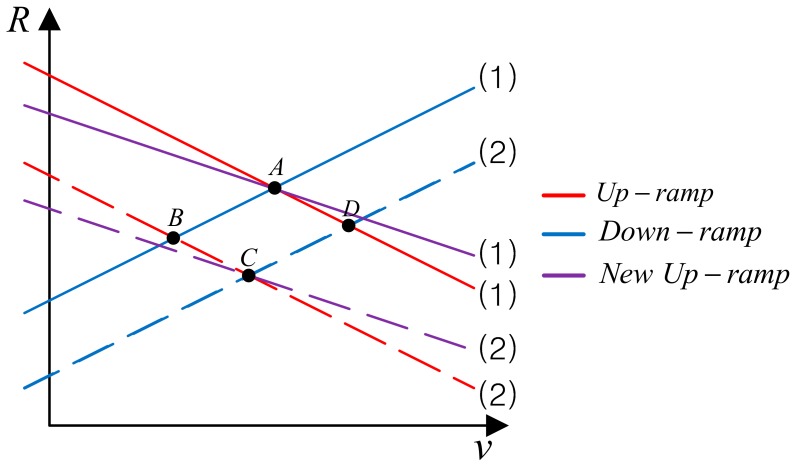
Detection of true target by adding a waveform with different slope.

To differentiate the true target from the ghost one, sometimes the system adds additional chirp signals with different slope ($\pm f_{c}T/B$) to the existing waveform [15,16,17]. This adds additional line with different slope in *R-v* plane as shown in Figure 4. By detecting the intersection points of the 3 lines, we can differentiate the true targets (A and C) from the ghost ones (B and D). One thing to mention is that though we explained the algorithm assuming the triangular waveform here, we can use a ramp waveform with different frequency slopes which will be shown in the following section.

## 3. Proposed Method

Currently there exists no regulation or coordination of the radar waveforms of autonomous vehicles. As the number of vehicles deploying radar sensors increases, it is inevitable to have radar waveform interferences between neighboring vehicles. The problem caused by the radar signal interference is similar to the multi-target problem described in the previous section. In this section we propose a novel method where vehicles choose a signal from a set of waveforms with different frequency domain slopes (±B/T).

**Figure 5:**
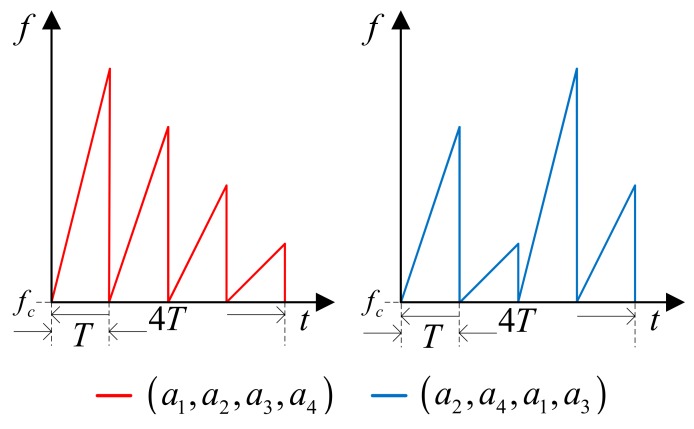
Example of the waveform of the generated waveform set.

In the proposed method, a single period of chirp sequence is composed of four short chirp sequences with different frequency slopes. Figure 5 shows an example of two possible chirp sequences. To minimize the interference between the radars of adjacent vehicles, which might not be time synchronized, we impose two constraints in choosing the slope sequences. First, we do not allow any two slope indices match in the same time interval. For example, if $\left(a_{1},\;a_{2},\;a_{3},\;a_{4}\right)$ is a possible slope sequence then $\left(a_{1},\;a_{3},\;a_{4},\;a_{2}\right)$ is not allowed since $a_{1}$ occupies the same slot. Second, we do not allow any cyclic permutation of a given slope sequence. For example, if $\left(a_{1},\;a_{2},\;a_{3},\;a_{4}\right)$ is a possible slope sequence then $\left(a_{2},\;a_{3},\;a_{4},\;a_{1}\right)$ is not allowed since the delayed waveform of the transmitted sequence $\left(a_{1},\;a_{2},\;a_{3},\;a_{4}\right)$ coincides with the second one. The two sequences in Figure 5 do not violate these constraints.

The transmitted signal between the time interval of 0~*NT* can be described by:

(7) sTi(t)=Re{∑k=0 N−1ej2π(fc+aki/2⋅(t−kT))⋅(t−kT)⋅rect(t−(k+1/2)TT)},

where $s_{T}^{i}\left(t\right)$ denotes the transmitted signal of the vehicle using *i*-th slope sequence, $a_{k}^{i}$ is a *k*-th slope of chirp signal, *N* is the number of the slope sequence in a single chirping interval (four in Figure 5) and *T* is the time duration of a short chirp sequence. The received signal can be described by:

(8) $$s_{R}(t)=\sum_{n=1\;}^{L_{i}}A_{n}s_{T}^{i}\left(t-\tau_{n}\right)+\sum_{m=1}^{M}\sum_{l_{m}=1}^{L_{m}}B_{m}^{l}s_{T}^{i_{m}}\left(t-\tau_{l_{m}}\right)+w\left(t\right),$$

where $A_{n}$, $B_{m}^{l}$ are attenuation coefficients, $\tau_{n}$ is the time delay, *M* is the number of interfering signals, *i_m_* is the slope sequence index, *L_i_* and *L_m_* are the number of the targets and interferences, and *w*(*t*) is the AWGN signal. That is, the first part is from the true targets and the second part is from the interferences.

Then, the signal processing process shown in Figure 1 is performed using Equations (7) and (8) resulting in the following output signal after mixing $s_{R}\left(t\right)$ and $s_{T}\left(t\right)$, as is given by:

(9) LPF{sTi(t)⋅sR(t) }=sb(t)+ib(t)+n(t)=Re{∑n=1Li(∑k=0N−1Anej2π(aki⋅τn−fdn)⋅(t−kT−τn)⋅rect(t−((k+1/2)T+τn/2)T−τn))+∑m=1M∑lm=1Lm(∑k=0N−1Bmlej2π(((aki−akim2)⋅(t−kT)+akim⋅τlm−fdm)⋅(t−kT−τlm))⋅rect(t−((k+1/2)T+τlm/2)T−τlm))}+n(t),

where $s_{b}\left(t\right)$,$\;i_{b}\left(t\right)$ are the signal and interference beat signal, respectively, and n(t) is the AWGN signal.

To obtain the beat frequencies of the targets, we apply the CFAR algorithm to each of the *N* short chirp sequences. The number of detected beat frequencies can be different in each sequence. Let $N_{k}$ denote the number of beat frequencies extracted in the *k*-th short sequence where *k* can be 1 to *N*. The process of detecting true targets using the beat frequencies extracted from CFAR is composed of the following three steps. First, to generate candidates for the true targets, we draw straight lines on the *R-v* plane using beat frequencies of two periods with the largest $N_{k}$ value. The reason for using the beat frequency of the period with the largest $N_{k}$ value is to increase the detection probability. This can also increase the false alarm rate, but this problem can be amended by the second step. If the *k* values at this time are expressed as $k_{1}$ and $k_{2}$, the straight lines on the R-v plane expressed using Equation (6) are expressed by:

(10) $$\{\begin{array}{c} R_{ij\;}=-f_{c}\frac{T}{B_{k_{1}}}v_{ij}+f_{i}^{k_{1}}\frac{cT}{2B_{k_{1}}}\\ R_{ij}=-f_{c}\frac{T}{B_{k_{2}}}v_{ij}+f_{j}^{k_{2}}\frac{cT}{2B_{k_{2}}} \end{array},$$

where $f_{i}^{k}$, $B_{k}$ refers to the *i*-th beat frequency and bandwidth of the *k*-th short sequence, respectively. So, the *R* and *v* profiles of the true target candidates using Equation (10) are expressed by:

(11) $$R_{ij\;}=\frac{cT}{2}\cdot \frac{f_{i}^{k_{1}}-f_{j}^{k_{2}}}{B_{k_{1}}-B_{k_{2}}},\;v_{ij}=\frac{c}{2f_{c}}\cdot \frac{f_{j}^{k_{2}}B_{k_{1}}-f_{i}^{k_{1}}B_{k_{2}}}{B_{k_{1}}-B_{k_{2}}}\;,$$

where $k_{1},k_{2}\in \left\{1,2,\ldots ,N\right\}$ and $i\in \left\{1,2,\ldots ,N_{k_{1}}\right\},j\in \left\{1,2,\ldots ,N_{k_{2}}\right\}$.

In the second step, we determine the true target by measuring the intersection points of *N* lines. Since there exists noise or other interference the targets do not meet at one point so we have to measure how close those crossing points are located. For this we measure the distances between the $N_{k_{1}}\times N_{k_{2}}$ target candidates (that is the combination of the two sequences chosen in the first step) and the straight lines of all the remaining sequences. Since the true target is expected to meet at one point if there exists no noise, we can assume that the true target has a small accumulated distance value.

Since a single chirp sequence is composed of multiple short chirp sequences, there could be different number of beat frequencies available (or many different $N_{k}$’s.). For this purpose, in the third step, the value $N_{k}$ of the period with the smallest INR (interference-to-noise ratio) value is set as the total number of true targets and denote the value as $N_{T}$. In this paper, we define $INR_{dB}=10\log_{10}\left(\frac{P_{Interference}}{P_{AWGN}}\right)$ where $P_{Interference}$ and $P_{AWGN}$ are signal power of the interference and AWGN. So, in the final step, $N_{T}$ candidates with the smallest average distance among the true target candidates are regarded as true ones.

When the slope sequence of the neighboring vehicles differs, the frequency of the beat signal varies with time as shown by:

(12) $$freq\left(i_{b}(t)\;\right)=2\pi \frac{d}{dt}\left(\left(\left(\frac{a_{k}^{i}-a_{k}^{i_{m}}}{2}\right)\cdot \left(t-kT\right)+a_{k}^{i_{m}}\cdot \tau_{l_{m}}-f_{d_{m}}\right)\cdot \left(t-kT-\tau_{l_{m}}\right)\right).$$

**Figure 6:**
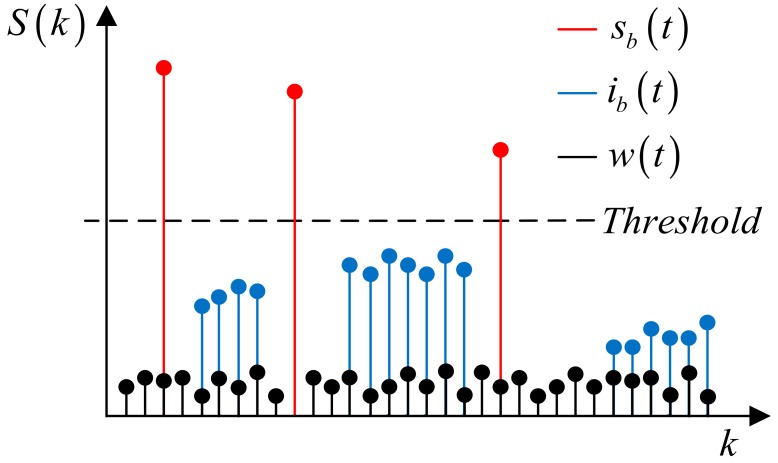
Fast Fourier Transform result of Equation (9). ($i\neq i_{m}$).

If i and $i_{m}$ are equal in Equation (12), the frequency of $i_{b}\left(t\right)$ can be represented as $\left(a_{k}^{i_{m}}\cdot \tau_{l_{m}}-f_{d_{m}}\right)$ which is a fixed frequency, it gives a peak in frequency domain which can be easily detected by CFAR algorithm. Otherwise, $freq\left(i_{b}\left(t\right)\right)$ become a function of time *t*, so it spreads out in the frequency domain, so it is not detected. In Figure 6, we show the case where 3 targets exist, and several interference signals are spread out in the FFT domain, so it is under the threshold of the CFAR detector.

Even though the slope sequence satisfies the two constraints explained before, if the transmission time of the two vehicles is not synchronized the slope sequence can overlap in time. For example, if the two slope sequences $\left(a_{1},\;a_{2},\;a_{3},\;a_{4}\right)$ and $\left(a_{2},\;a_{4},\;a_{1},\;a_{3}\right)$ are chosen but the latter sequence starts *T* second later then $a_{2}$ sequence overlaps. In this case, the interference signal corresponding to the period in which the slope overlaps is not removed. However, since the interference of other periods is eliminated, the performance does not degrade much.

## 4. Simulation Results

**Figure 7:**
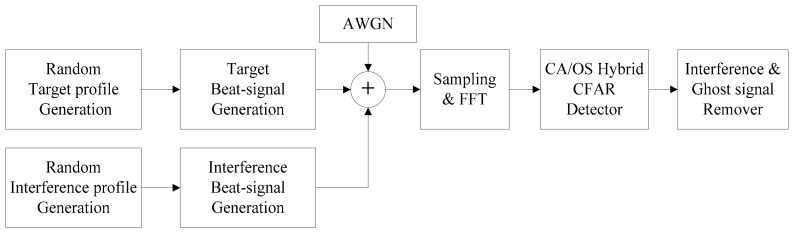
FMCW radar simulation block diagram.

The block diagram of the radar simulation is shown in Figure 7. In this simulation we consider the environment where five targets exist, and six interference signals are mixed with the true target signal. Each target and interference were randomly generated within the range of 0–150 m and relative velocity was randomly set between −100 m/s (−360 km/h)–50 m/s (180 km/h). The simulation parameters are shown in Table 1. The SNR of the AWGN versus the beat signal of the target used in the simulation was calculated based on the path loss model of reference [2]. We use a hybrid of OS-CFAR and CA-CFAR algorithm to improve the detection accuracy [18].

In the simulation, a single period of a chirp sequence is composed of four short chirp sequences with different frequency slopes. Each short chirp sequence lasts 0.5 ms (the total chirp sequence is 2 ms) with the frequency slopes selected from the four different values of ±1200, ±900, ±600, ±300 MHz/msec. If we do not allow the overlap of the same frequency slopes in the same short chirp interval, only eight short chirp sequence patterns are available as shown in Table 2. All short chirp sequences except the short chirp sequence of Table 2 do not satisfy the constraint for choosing the short chirp sequence described above. To verify the performance of the proposed method, we compare with the case where all the vehicles share the same short chirp sequences (which we denote as a conventional method) and where each vehicle use their own chirp sequence (which we denote as a proposed method).

Before, we defined INR as an interference to noise ratio, which is the power ratio between the total interference and AWGN signal in dB. We compared the performance of the proposed method and the conventional method in terms of detection probability and the number of false alarms while varying the INR value from −30 dB to 0 dB. The INR range is determined based on the real traffic environment. Though the INR 0 dB may seem small, however, since interference is concentrated in a specific frequency components, it is not small compared with the transmitted signal. Each simulation was performed 10,000 times and the average value was compared in the comparisons.

**Figure 8:**
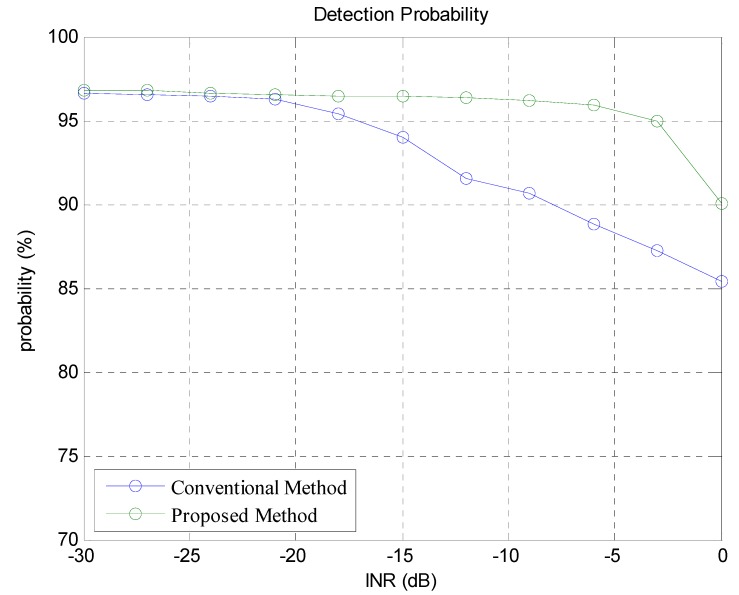
Detection probability of the proposed and conventional methods.

In Figure 8, we have shown the detection probability of the true target in comparison with the conventional method. In this paper, we define detection probability as $P_{D}\left(\%\right)=\frac{\mathrm{Number}\;\mathrm{of}\;\mathrm{matching}\;\mathrm{targets}}{\mathrm{Number}\;\mathrm{of}\;\mathrm{total}\;\mathrm{true}\;\mathrm{targets}}\times 100$. Here, the matching target means a detected target whose range and velocity difference with the true target is below a certain threshold. In Figure 8, the *x*-axis represents the INR defined in the previous section and *y*-axis represents the detection probability. When the INR value is less than −20 dB, the effect of interference is very small, so the difference of the detection probability between the proposed and conventional method is small. When the INR is greater than −20 dB, the detection probability of the conventional method decreases whereas that of the proposed method hardly changes as the INR increases. As INR approaches 0 dB, the interference signal power increases significantly compared with the signal power from the true target. In this case the detection probability of both the two methods decreases, however, the proposed system still outperforms the conventional one.

**Figure 9:**
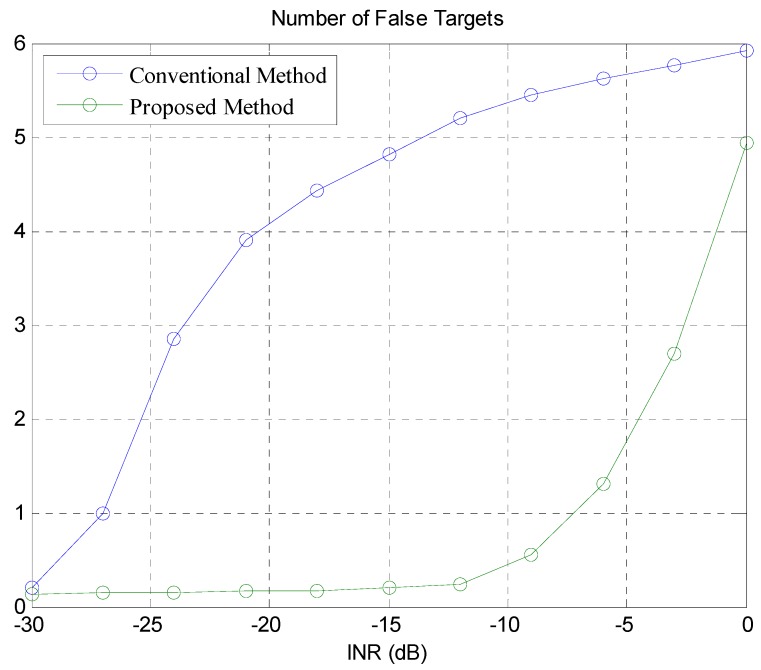
Average number of the false targets of the proposed and conventional methods.

In Figure 9, we show the average number of the false targets detected as we increase the INR. When the INR value is −30 dB, the performance of the proposed method is very similar to that of the conventional method in terms of false alarms. As the INR value increases the number of false targets increases accordingly. However, the number of the false targets of the conventional method is significantly larger than the proposed method. In the proposed method, though the INR value increases up to −10 dB, the interference is filtered through the CFAR, and the false alarm rarely occurs. If the INR value is greater than −10 dB, the number of false alarms generated in the proposed method starts to increase. However, the performance of the proposed method is still much better than the conventional method. This shows that the proposed method effectively removes the false targets unlike the conventional method which is susceptible to the interference generated by other neighboring vehicles.

## 5. Conclusions

Vehicles equipped with radar sensors to support ADAS are becoming widespread. As the number of the radar sensors increases, mutual radar-to-radar signal interference is becoming a serious problem causing the ghost-target problem. In this paper we propose a novel method to reduce the radar signal interference, where each vehicle uses a FMCW chirp signal whose frequency slope is different from that of the neighboring vehicles. Simulation results show that the detection probability and the number of false alarms performance is improved significantly by the proposed method.

## Figures and Tables

**Table 1:** Simulation parameters.

Specification	Value
**Carrier Frequency (*f_c_*)**	77 GHz
**Sampling Frequency (*F_s_*)**	10 MHz
**FFT point**	8192
**CFAR window**	16
**CA-CFAR Guard Cell**	2
**OS-CFAR Rank Selection (*r*)**	4

**Table 2:** Example of chirp sequence patterns.

Chirp Sequences (MHz/ms)
(1200, 900, 600, 300)
(900, 300, 1200, 600)
(600, 1200, 300, 900)
(300, 600, 900, 1200)
−(1200, 900, 600, 300)
−(900, 300, 1200, 600)
−(600, 1200, 300, 900)
−(300, 600, 900, 1200)
